# Recurrence and Mortality Risks in Patients with First Incident Acute Stroke or Myocardial Infarction: A Longitudinal Study Using the Korean National Health Insurance Service Database

**DOI:** 10.3390/jcm12020568

**Published:** 2023-01-10

**Authors:** Dougho Park, Mun-Chul Kim, Daeyoung Hong, Yong-Suk Jeong, Hyoung Seop Kim, Jong Hun Kim

**Affiliations:** 1 Department of Rehabilitation Medicine, Pohang Stroke and Spine Hospital, Pohang 37659, Republic of Korea; 2 Department of Medical Science and Engineering, School of Convergence Science and Technology, Pohang University of Science and Technology, Pohang 37673, Republic of Korea; 3 Department of Neurosurgery, Pohang Stroke and Spine Hospital, Pohang 37659, Republic of Korea; 4 Department of Cardiology, Pohang Stroke and Spine Hospital, Pohang 37659, Republic of Korea; 5 Department of Physical Medicine and Rehabilitation, National Health Insurance Service Ilsan Hospital, Goyang 10444, Republic of Korea; 6 Department of Neurology, National Health Insurance Service Ilsan Hospital, Goyang 10444, Republic of Korea

**Keywords:** epidemiology, mortality, myocardial infarction, national health programs, stroke

## Abstract

Background: We aimed to identify the long-term risk of recurrence and mortality in patients who experienced acute ischemic stroke (AIS), acute myocardial infarction (AMI), or acute hemorrhagic stroke (AHS) using a population-level database. Methods: This retrospective cohort study included adults aged ≥55 years diagnosed with AIS, AMI, and AHS in the National Health Insurance Service Database between 2004 and 2007. The target outcomes were secondary AIS, AMI, AHS, and all-cause mortality. Predetermined covariates, such as age, sex, socioeconomic status, hypertension, diabetes, and dyslipidemia, were adjusted. Results: We included 151,181, 49,077, and 41,636 patients in the AIS, AHS, and AMI groups, respectively. The AMI (adjusted hazard ratio [aHR], 0.318; 95% confidence interval [CI], 0.306–0.330; *p* < 0.001) and AHS (aHR, 0.489; 95% CI, 0.472–0.506; *p* < 0.001) groups had a significantly lower risk of developing secondary AIS than the AIS group. The risk of developing secondary AMI was significantly lower in the AMI (aHR, 0.388; 95% CI, 0.348–0.433; *p* < 0.001) and AHS (aHR, 0.711; 95% CI, 0.640–0.790; *p* < 0.001) groups than in the AIS group. Initial AHS was a decisive risk factor for secondary AHS (aHR, 8.546; 95% CI, 8.218–8.887; *p* < 0.001). The AMI (aHR, 1.436; 95% CI, 1.412–1.461; *p* < 0.001) and AHS (aHR, 1.328; 95% CI, 1.309–1.348; *p* < 0.001) groups were associated with a significantly higher risk of long-term mortality than the AIS group. Conclusion: Our results elucidated that initial AIS was a significant risk factor for recurrent AIS and AMI; initial AHS was a decisive risk factor for developing secondary AHS. Further, AMI and AHS were more closely related to long-term mortality than AIS.

## 1. Introduction

Stroke is associated with high morbidity and mortality rates worldwide [1]. In South Korea, cerebrovascular diseases were the fourth leading cause of death in 2021, with 44.0 deaths per 100,000 individuals [2]. In addition, 105,000 people experience their first incident or recurrent stroke every year in South Korea [3]. Acute ischemic stroke (AIS) accounts for most stroke cases; however, approximately 10–15% of stroke cases are acute hemorrhagic stroke (AHS) cases [4]. Long-term disabilities after acute stroke cause a decrease in an individual patient’s quality of life and an increase in social healthcare costs [5,6].

Cardiovascular disease has been the second leading cause of death in South Korea since 2014, accounting for 61.5 deaths per 100,000 people in 2021 [2]. Hospitalization due to cardiovascular diseases is also increasing in South Korea [7]. Most deaths from ischemic heart disease are caused by acute myocardial infarction (AMI) [8], which results in recurrence or other complications, and long-term deterioration of the patient’s quality of life due to reduced exercise capacity [2].

**Figure 1:**
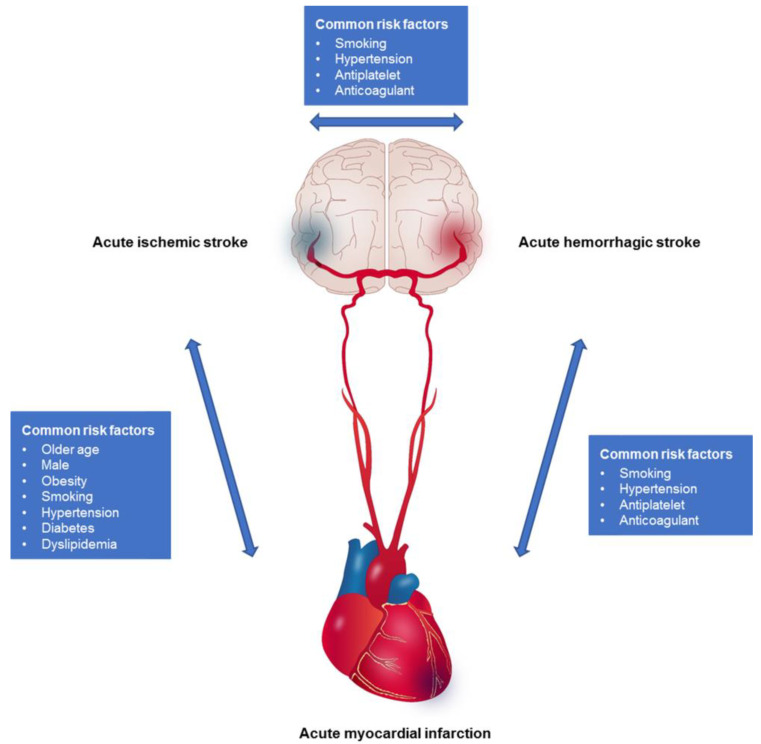
Interrelated brain–heart diseases and common risk.

Brain and heart diseases are interrelated and have been reported in previous studies [9]. AIS and AMI share several risk factors [10]. In addition to comorbidities, such as hypertension, diabetes, and dyslipidemia, demographic factors, such as age and sex, and behavioral factors (e.g., smoking and obesity) are also known as common risk factors [11,12,13]. Although AHS has different pathophysiological risk factors, few risk factors, such as hypertension and smoking, are associated with the development of ischemic events [14,15]. Medications for secondary prevention after ischemic events are another risk factor for AHS [16]. Therefore, the primary event acts as a risk factor and affects the mutual recurrence of acute stroke and myocardial infarction events (Figure 1).

Acute stroke and AMI are significant causes of death in South Korea and require plenty of medical resources [17,18,19]. Therefore, to prevent primary and secondary cardio-cerebrovascular diseases and efficiently distribute related medical resources, it is necessary to investigate the epidemiological features of acute stroke and AMI. However, studies on the mutual risk of recurrence for each cardio-cerebrovascular event in South Korea are lacking.

In this study, we investigated long-term risks of recurrence and mortality in patients with AIS, AMI, and AHS, using a population-based survey in South Korea. Thus, we aimed to provide epidemiologic background data on major cardio-cerebrovascular events reported in the national public health system.

## 2. Materials and Methods

### 2.1. Participants and Study Design

**Figure 2:**
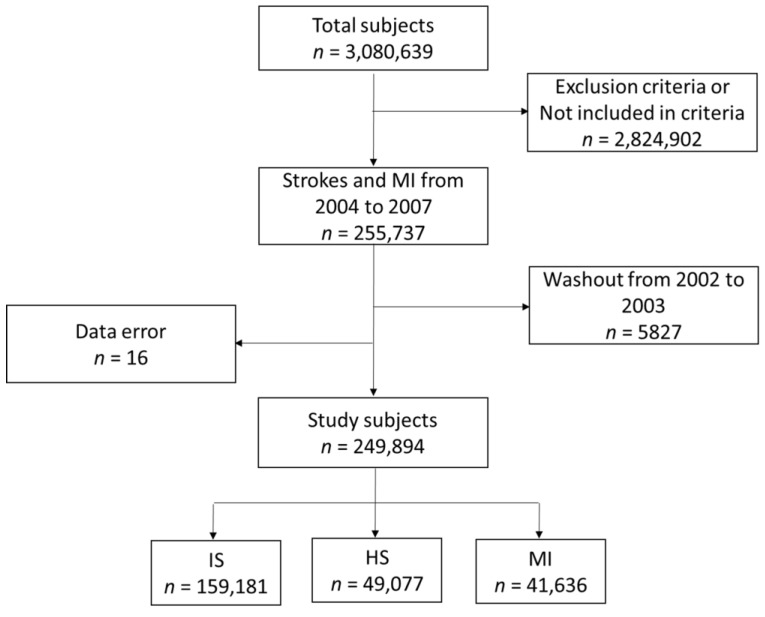
Flowchart of patient inclusion. ICD, international classification of diseases; Dx, diagnosis; MI, myocardial infarction.

This was a retrospective cohort study. The initial cohort was defined as adults aged ≥55 years diagnosed with AIS, AMI, and AHS in the National Health Insurance Service (NHIS) database from 2004 to 2007. A total of 303,596 participants were initially enrolled. Patients with underlying hemorrhagic or thrombotic conditions were excluded (*n* = 15,601). A detailed explanation of the underlying hemorrhagic and thrombotic conditions is provided in Table S1. Furthermore, we excluded patients who did not meet the diagnostic criteria for AIS, AMI, or AHS, as described in the following section. To extract the first incident event, a washout period from 2002 to 2003 was set (*n* = 5827) and 16 participants with data errors were excluded. Finally, 249,894 participants were included in the study (Figure 2). We observed patients until December 2019 and established death, recurrent AIS, AHS, or AMI, and the first occurrence after the initial event, as the primary outcomes. Outcome events that occurred within 30 days of the initial event were excluded. The Institutional Review Board of the NHIS Ilsan Hospital reviewed and approved this study. Informed consent was waived because of the retrospective study design (approval number: 2022-11-027). This study was conducted in accordance with the principles of the Declaration of Helsinki.

### 2.2. Variable Definitions

The participants’ age, sex, and socioeconomic status were identified as demographic variables. We defined the patient’s socioeconomic status (SES) as the level of household income divided by national health insurance premiums into 20 quartiles: individuals with low, middle, and high SES were defined as those being in the ≤7th, 8–14th, and ≥15th quartiles, respectively. Hypertension, diabetes, and dyslipidemia were identified as comorbidities. Hypertension was identified as the use of anti-hypertensive medication or systolic blood pressure ≥140 mmHg or diastolic blood pressure ≥ 90 mmHg in the medical examination data. Diabetes was identified as using oral hypoglycemic agents or having a fasting blood glucose level of ≥7.0 mmol/L at the time of medical examination. Finally, dyslipidemia was defined as taking lipid-lowering agents or having a total cholesterol level of >200 mg/dL at the time of medical examination.

Major cardio-cerebrovascular events were defined using the International Classification of Disease-10 codes. First, AIS was defined as hospitalization with an I63 code and brain-computed tomography or magnetic resonance imaging within 7 days following hospitalization. AHS was defined as hospitalization with I60–I62 diagnostic codes and in cases where brain-computed tomography or magnetic resonance imaging was performed within 7 days of hospitalization. For AMI, in cases of hospitalization with I21–I23 codes and two or more requests for creatine kinase myocardial band and troponin tests, one or more coronary angiographies (CAG) at index admission or death within 1 month of admission were identified. Finally, we identified all-cause mortality during the follow-up period.

### 2.3. Statistical Analysis

Continuous variables were expressed as means ± standard deviations; one-way analysis of covariates with Bonferroni correction was used for group comparisons. Categorical variables were expressed as frequencies and proportions and analyzed using the chi-square test. Kaplan–Meier estimation was used to analyze the cumulative probability of mortality and recurrent events univariably. Multivariable Cox regression analysis was used to analyze the mutual risk of recurrence and mortality between major cardio-cerebrovascular events. In the Cox proportional hazards models, predetermined covariates, such as age, sex, SES, hypertension, diabetes, and dyslipidemia were adjusted. We established Cox proportional hazards models to treat the competing risks between the major cardio-cerebrovascular events and death using the Fine and Gray models. Statistical significance was set at *p* < 0.05. All statistical analyses were performed using the SAS Enterprise Guide 7.15 (SAS Institute, Cary, NC, USA). R (version 4.0.5; R Foundation for Statistical Computing, Vienna, Austria) was used to create Kaplan–Meier graphs.

## 3. Results

### 3.1. Baseline Characteristics

The baseline characteristics of each initial event group are presented in Table 1. We included 151, 181, 49,077, and 41,636 patients in the AIS, AHS, and AMI groups, respectively. The mean follow-up period was 7.23 ± 5.70 years for AIS, AMI, and AHS and 8.47 ± 5.59 years for mortality. The number of cases and baseline characteristics of each secondary event in each group are presented in Tables S2–S4.

### 3.2. Cox Proportional Hazards Models

#### 3.2.1. Secondary Acute Ischemic Stroke

**Figure 3:**
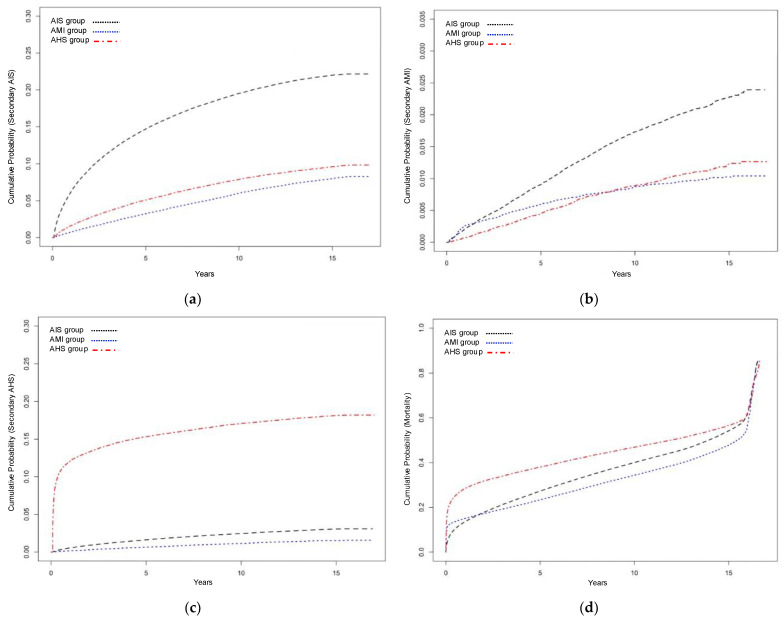
Kaplan–Meier curves indicating the cumulative probability of developing secondary (**a**) AIS, (**b**) AMI, (**c**) and AHS, and (**d**) mortality. AIS, acute ischemic stroke; AMI, acute myocardial infarction; AHS, acute hemorrhagic stroke.

The AIS group showed a higher cumulative probability of developing secondary AIS than the AMI and AHS groups (Figure 3a). Cox regression analysis showed that the AMI (adjusted hazard ratio [aHR], 0.318; 95% confidence interval [CI], 0.306–0.330; *p* < 0.001) and AHS (aHR, 0.489; 95% CI, 0.472–0.506; *p* < 0.001) groups had a significantly lower risk of secondary AIS than the AIS group (Table 2).

#### 3.2.2. Secondary Acute Myocardial Infarction

In the case of secondary AMI, the initial AMI group had a slightly higher cumulative probability than the AIS group within 2 years. However, the cumulative probability was higher in the AIS group in the long term (Figure 3b). Cox regression analysis confirmed that the risk of developing secondary AMI was significantly lower in the AMI (aHR, 0.388; 95% CI, 0.348–0.433; *p* < 0.001) and AHS (aHR, 0.711; 95% CI, 0.640–0.790; *p* < 0.001) groups than in the AIS group (Table 3).

#### 3.2.3. Secondary Acute Hemorrhagic Stroke

Secondary AHS had a significantly higher cumulative probability in the initial AHS group, and it was confirmed that most events occurred within 2 years after the initial AHS (Figure 3c). Cox regression analysis identified the AHS group as an independent factor with the highest risk of secondary AHS (aHR, 8.546; 95% CI, 8.218–8.887; *p* < 0.001). In contrast, the AMI group had a significantly lower risk of developing secondary AHS than the AIS group (aHR, 0.555; 95% CI, 0.508–0.607; *p* < 0.001) (Table 4).

#### 3.2.4. Long-Term Mortality

The cumulative probability for mortality was the highest in the AHS group. In the case of ischemic events, the cumulative death rate in the AMI group was slightly higher at the beginning. However, the cumulative mortality rate of the AIS group was relatively higher, approximately 2 years after the initial events (Figure 3d). In the Cox regression analysis adjusted for other covariates, the AMI (aHR, 1.436; 95% CI, 1.412–1.461; *p* < 0.001) and AHS (aHR, 1.328; 95% CI, 1.309–1.348; *p* < 0.001) groups were associated with a significantly higher risk of long-term mortality than the AIS group (Table 5).

## 4. Discussion

In this study, we reported the long-term risk of recurrence and mortality in patients with initial AIS, AMI, and AHS, using a large NHIS database cohort. We confirmed that initial AIS was a significant risk factor for recurrent AIS and AMI and that initial AHS was a decisive risk factor for secondary AHS. Unlike previous studies, which typically relied on small sample sizes, this study confirmed the long-term risk of recurrence and mortality among patients with AIS, AMI, and AHS using extensive population-based data. Furthermore, we provided an epidemiological basis for initial and recurrent major cardio-cerebrovascular events in the Korean NHIS.

Previous studies have reported a mutual risk between cardio-cerebrovascular diseases [20,21,22]. In particular, many cases of AIS and AMI share a systematic condition called atherosclerosis [23]. In particular, 2–6% of deaths within the first 3 months in the AIS patient group were reported as cardiac causes [24], and asymptomatic stenosis in CAG in patients with AIS was found to be a strong predictor of major vascular events in 2 years [25]. Therefore, patients with AIS, whose pathologies are primarily atherosclerotic or cardioembolic sources should undergo cardiac assessments, including coronary artery evaluation [26]. Kang et al. [27] studied the occurrence of recurrent AIS and AMI within 1 year after AIS using a Korean multicenter registry. They reported that AIS recurred in 5.7% of patients, and 13.7% of patients experienced major vascular events, most of which occurred within 90 days. Lee et al. [28] also used a Korean multicenter database to track AMI risk after AIS for 5 years. In their study, the 5-year cumulative AMI incidence was 2.0% and prior coronary heart disease was the most potent risk factor for AMI after AIS. Our study observed relevant events longer than those reported in the previous two studies and had the distinct advantage of minimizing case loss using NHIS claim data. In addition, our results confirmed that AIS was a decisive risk factor for secondary ischemic events (AIS and AMI), which is consistent with the results of previous studies [29]. We deduced that one of the reasons for these results was that, in the AMI patient group, many patients with higher severity died within 3 months, and relatively mild-to-moderate patients were enrolled in the final cohort.

In this study, the participants in the AMI group were the youngest, which is consistent with the results of previous studies [30]. In addition, AMI had the highest proportion of male patients, as reported in previous studies [31]. There have been reports of AIS occurrence after an initial AMI. The incidence of AIS within 1 year of AMI was reported to be 3.7%, most of which occurs in the acute phase [32,33]. In addition, it has been reported that the mortality rate of patients with initial AMI accompanied by secondary AIS is approximately 30% [34]. Although recent studies have suggested that the risk of AIS in the AMI group tended to decrease due to the development of appropriate early revascularization and medications and the early screening of the cerebrovascular system [35], when AIS occurs in patients with AMI, the prognosis was worse, which is consistent with many studies [24]. In this study, the AMI group had a higher long-term mortality risk than the AIS group, which was attributed to heterogeneous mechanisms of stroke.

Our results confirmed that prior AHS was the strongest risk factor for secondary AHS, which is consistent with the results of previous studies [36]. The long-term risk evaluation of AIS and AMI secondary to AHS remains poorly understood. Murthy et al. [37] reported a rate of developing arterial ischemic events of 5.7% within 1 year in patients with intracranial hemorrhage, with most events occurring within the first 6 months. In addition, Casolla et al. [38] reported that 20% of the patients with intracranial hemorrhage experienced an incident ischemic event. Previous studies have reported that most cases of AHS after AMI occur following acute thrombolysis [39]. Moreover, relatively long-term studies that have observed the occurrence of AHS after AMI are scarce. We verified the trend of long-term ischemic events in a relatively large number of patients with AHS. Similar to AMI, it was confirmed that long-term mortality risk was significantly higher in the AHS than in the AIS group, which is consistent with previous studies [40]. Consequently, although they share some risk factors, AHS is heterogeneous compared to other ischemic events, and further epidemiologic studies of major vascular events following AHS are required to elucidate their association.

Among the covariates used in our study, it was observed that hypertension and hyperlipidemia were related to favorable outcomes, unlike the results of previous studies [41]. The authors suggest the following reasons for these results: first, we inferred that these results were related to health-seeking behaviors; patients regularly receiving chronic disease management would detect minor vascular events early and be treated more appropriately [42]. Under the NHIS system in South Korea, chronic diseases, such as hypertension, diabetes, and dyslipidemia, can be managed with relatively low medical costs and with better medical access. However, our definitions of hypertension and dyslipidemia may be highly sensitive. This could be inferred from the high rate of hypertension in our cohort. In contrast, factors, such as lower SES, male sex, and age, increased the risk of ischemic events and mortality in this study, consistent with the findings of previous studies [43,44].

### Strengths and Limitations

As we used claims data from the NHIS, we are confident that almost all events could be objectively screened using the diagnostic codes of target outcomes. In addition, this study presents long-term follow-up data on relevant cardio-cerebrovascular diseases based on a large population-level sample. We excluded secondary events that occurred within 30 days of the first event; therefore, we could only report the long-term risks of recurrence and mortality to a certain extent. Finally, our study provides multifaceted epidemiological features by reciprocal analysis of the long-term risks between AIS, AMI, AIS, and mortality.

However, this study had several limitations. As we retrospectively analyzed population-based data, it was challenging to clarify the severity and detailed pathophysiological features of the observed cardio-cerebrovascular events. Stroke mechanisms are quite heterogeneous compared to those of AMI and show various manifestations depending on the cause. There are various causes of AIS, such as large-artery atherosclerosis, cardioembolic source, or small vessel occlusion, and there may be differences in severity and disease course in each case. In the case of AHS, it was difficult to reflect the characteristics according to the hemorrhage types. In our cohort, both subarachnoid and intracranial hemorrhage were included in the AHS group. Similarly, in the AMI group, features such as left ventricular hypertrophy could not be further adjusted, which was a limitation of our study. In addition, this study did not define underlying conditions, such as family history, smoking, alcohol consumption, and medication history. The fact that we did not consider the preventive effect of medications on ischemic events, in which secondary prevention is important, was a major limitation of this study. We enrolled patients aged ≥55 years to focus on the high-risk age groups. However, this may have led to selection bias in the results. Finally, this study identified all-cause mortality instead of death by cardio- or cerebrovascular events due to the limitations of our database.

## 5. Conclusions

Using population-level data from South Korea, this study comprehensively investigated the long-term risk of secondary events and mortality after AIS, AMI, and AHS. We found that AIS significantly increased the long-term risk of developing secondary AIS and AMI events, and AHS was the strongest risk factor for secondary AHS. These cardio-cerebrovascular diseases were interrelated; however, AHS showed different characteristics. This study provides an epidemiological basis for recurrent major cardiocerebrovascular events in the Korean NHIS. Further, future research with detailed disease-specific clinical information is needed.

## Figures and Tables

**Table 1:** Baseline characteristics.

Variables	AIS Group (*n* = 159,181)	AMI Group (*n* = 41,636)	AHS Group (*n* = 49,077)	*p* Value
Age, years	68.5 ± 11.5	64.3 ± 12.2	67.6 ± 11.2	<0.001
Male, *n* (%)	81,047 (50.9)	28,008 (67.3)	23,064 (47.0)	<0.001
Diabetes, *n* (%)	46,152 (29.0)	12,976 (31.2)	7547 (15.4)	<0.001
Hypertension, *n* (%)	149,622 (94.0)	40,013 (96.1)	41,557 (84.7)	<0.001
Dyslipidemia, *n* (%)	76,647 (48.2)	32,643 (78.4)	13,177 (26.8)	<0.001
SES, *n* (%)				<0.001
Low	53,184 (33.4)	11,971 (28.8)	15,442 (31.5)	
Middle	47,244 (29.7)	13,209 (31.7)	15,665 (31.9)	
High	58,753 (36.9)	16,456 (39.5)	17,970 (36.6)	

AIS, acute ischemic stroke; AMI, acute myocardial infarction; AHS, acute hemorrhagic stroke; SES, socioeconomic status.

**Table 2:** Cox proportional hazards model for secondary acute ischemic stroke.

Variables	Adjusted HR	95% Confidence Interval	*p* Value
Initial event			
AIS	1.000		
AMI	0.318	0.306–0.330	<0.001
AHS ^a^	0.489	0.472–0.506	<0.001
Comorbidities			
Diabetes	1.310	1.283–1.338	<0.001
Hypertension	1.026	0.970–1.085	0.377
Dyslipidemia	0.928	0.909–0.948	<0.001
SES			
Low	1.000		
Middle	0.883	0.862–0.905	<0.001
High	0.837	0.817–0.857	<0.001
Age (per year)	1.018	1.017–1.019	<0.001
Female	0.865	0.847–0.883	<0.001

^a^ The AHS group showed a significantly higher risk of developing secondary AIS than the AMI group. HR, hazard ratio; AIS, acute ischemic stroke; AMI, acute myocardial infarction; AHS, acute hemorrhagic stroke; SES, socioeconomic status.

**Table 3:** Cox proportional hazards model for secondary acute myocardial infarction.

Variables	Adjusted HR	95% Confidence Interval	*p* Value
Initial event			
AIS	1.000		
AMI	0.388	0.348–0.433	<0.001
AHS ^a^	0.711	0.640–0.790	<0.001
Comorbidities			
Diabetes	1.666	1.555–1.785	<0.001
Hypertension	0.796	0.657–0.965	0.020
Dyslipidemia	1.221	1.136–1.313	<0.001
SES			
Low	1.000		
Middle	0.939	0.864–1.020	0.137
High	0.901	0.832–0.976	0.011
Age (per year)	1.013	1.010–1.017	<0.001
Female	0.625	0.582–0.671	<0.001

^a^ The AHS group showed a significantly higher risk of developing secondary AMI than the AMI group. HR, hazard ratio; AIS, acute ischemic stroke; AMI, acute myocardial infarction; AHS, acute hemorrhagic stroke; SES, socioeconomic status.

**Table 4:** Cox proportional hazard model for secondary acute hemorrhagic stroke.

Variables	Adjusted HR	95% Confidence Interval	*p* Value
Initial event			
AIS	1.000		
AMI	0.555	0.508–0.607	<0.001
AHS	8.546	8.218–8.887	<0.001
Comorbidities			
Diabetes	0.965	0.923–1.009	0.120
Hypertension	0.687	0.646–0.731	<0.001
Dyslipidemia	0.842	0.811–0.875	<0.001
SES			
Low	1.000		
Middle	1.005	0.962–1.049	0.838
High	1.013	0.971–1.056	0.550
Age (per year)	0.998	0.996–0.999	0.007
Female	1.132	1.092–1.173	<0.001

HR, hazard ratio; AIS, acute ischemic stroke; AMI, acute myocardial infarction; AHS, acute hemorrhagic stroke; SES, socioeconomic status.

**Table 5:** Cox proportional hazard model for mortality.

Variables	Adjusted HR	95% Confidence Interval	*p* Value
Initial event			
AIS	1.000		
AMI ^a^	1.436	1.412–1.461	<0.001
AHS	1.328	1.309–1.348	<0.001
Comorbidities			
Diabetes	1.339	1.321–1.357	<0.001
Hypertension	0.406	0.398–0.414	<0.001
Dyslipidemia	0.582	0.574–0.590	<0.001
SES			
Low	1.000		
Middle	0.939	0.926–0.953	<0.001
High	0.846	0.834–0.857	<0.001
Age (per year)	1.092	1.091–1.093	<0.001
Female	0.755	0.746–0.764	<0.001

^a^ The AMI group showed a significantly higher risk of mortality than the AHS group. HR, hazard ratio; AIS, acute ischemic stroke; AMI, acute myocardial infarction; AHS, acute hemorrhagic stroke; SES, socioeconomic status.

## Data Availability

The data from this study are not publicly available because of the privacy and ethical restrictions of the Korean National Health Insurance data sharing system. The dataset used in this study can only be accessed by an authorized researcher.

## Supplementary Materials

The following supporting information can be downloaded at: https://www.mdpi.com/article/10.3390/jcm12020568/s1, Table S1. List of underlying hemorrhagic and thrombotic conditions for exclusion; Table S2. Baseline characteristics according to the secondary events in patients with initial acute ischemic stroke; Table S3. Baseline characteristics according to the secondary events in patients with initial acute myocardial infarction; Table S4. Baseline characteristics according to the secondary events in patients with initial acute hemorrhagic stroke.
