# Supplementary material for: Recurrence and Mortality Risks in Patients with First Incident Acute Stroke or Myocardial Infarction: A Longitudinal Study Using the Korean National Health Insurance Service Database
Source: J Clin Med. 2023 Jan 10;12(2):568. doi: 10.3390/jcm12020568 (PMC9865804; doi:10.3390/jcm12020568)
Supplement: Supplementary file 1 [file jcm-12-00568-s001.zip › jcm-2146940-supplementary.pdf]

Supplementary Materials

Table S1. List of underlying hemorrhagic and thrombotic conditions for exclusion.

ICD-10 code	Description
**D45**	Polycythemia vera
**D47.3**	Essential (hemorrhagic) thrombocythemia
**D68.4**	Acquired coagulation factor deficiency
**G45.9**	Transient cerebral ischemic attack, unspecified
**H34.0**	Transient retinal artery occlusion
**H34.2**	Other retinal artery occlusions
**H34.9**	Unspecified retinal vascular occlusion
**I09.9**	Rheumatic heart disease, unspecified
**I20**	Angina pectoris
**I23.6**	Thrombosis of atrium, auricular appendage, and ventricle as current complications following acute myocardial infarction
**I24.0**	Acute coronary thrombosis not resulting in myocardial infarction
**I25**	Chronic ischemic heart disease
**I26.9**	Pulmonary embolism without acute cor pulmonale
**I48**	Atrial fibrillation and flutter
**I49.0**	Ventricular fibrillation and flutter
**I50**	Heart failure
**I50.1**	Left ventricular failure, unspecified
**I50.9**	Heart failure, unspecified
**I51.3**	Intracardiac thrombosis, not elsewhere classified
**I65.9**	Occlusion and stenosis of unspecified precerebral artery
**I66**	Occlusion and stenosis of cerebral arteries, not resulting in cerebral infarction
**I67.2**	Cerebral atherosclerosis
**I67.7**	Cerebral arteritis, not elsewhere classified
**I70.0**	Atherosclerosis of aorta
**I70.1**	Atherosclerosis of renal artery
**I74.0**	Embolism and thrombosis of abdominal aorta
**I74.1**	Embolism and thrombosis of other and unspecified parts of aorta
**I74.2**	Embolism and thrombosis of arteries of the upper extremities
**I74.3**	Embolism and thrombosis of arteries of the lower extremities
**I74.4**	Embolism and thrombosis of arteries of extremities, unspecified
**I74.5**	Embolism and thrombosis of iliac artery
**I74.8**	Embolism and thrombosis of other arteries
**I74.9**	Embolism and thrombosis of unspecified artery
**I97.1**	Other postprocedural cardiac functional disturbances
**K55.0**	Acute vascular disorders of intestine
**K55.1**	Chronic vascular disorders of intestine
**K70.2**	Alcoholic fibrosis and sclerosis of liver
**K70.3**	Alcoholic cirrhosis of liver
**K74.6**	Other and unspecified cirrhosis of liver
**N28.0**	Ischemia and infarction of kidney
**T86.2**	Complications of heart transplant
**T86.4**	Complications of liver transplant
**Z94.4**	Liver transplant status
**Z95.2**	Presence of prosthetic heart valve

Table S2. Baseline characteristics according to the secondary events in patients with initial acute ischemic stroke.

Variables	Secondary Events				
	Death (*n* = 73,300)	AIS (*n* = 33,491)	AMI (*n* = 2746)	AHS (*n* = 3886)	No Event (*n* = 45,758)
**Age, years**	74.3 ± 9.0	67.4 ± 10.8	65.9 ± 10.6	66.8 ± 10.4	60.4 ± 10.6
**Male, *n* (%)**	36,206 (49.4)	17,626 (52.6)	1702 (62.0)	2021 (52.0)	23,492 (51.3)
**Diabetes, *n* (%)**	21,242 (29.0)	11,287 (33.7)	1133 (41.3)	1044 (26.9)	11,446 (25.0)
**Hypertension, *n* (%)**	66,201 (90.3)	32,418 (96.8)	2661 (96.9)	3747 (96.4)	44,595 (97.5)
**Dyslipidemia, *n* (%)**	26,703 (36.4)	17,776 (53.1)	1702 (62.0)	1967 (50.6)	28,499 (62.3)
**SES, *n* (%)**					
**Low**	26,150 (35.7)	11,673 (34.9)	872 (31.8)	1250 (32.2)	13,239 (28.9)
**Middle**	20,206 (27.6)	9836 (29.4)	851 (31.0)	1225 (31.5)	15,126 (33.1)
**High**	26,944 (36.8)	11,982 (35.8)	1023 (37.3)	1411 (36.3)	17,393 (38.0)

Abbreviations: AHS, acute hemorrhagic stroke; AIS, acute ischemic stroke; AMI, acute myocardial infarction; SES, socioeconomic status.

Table S3. Baseline characteristics according to the secondary events in patients with initial acute myocardial infarction.

Variables	Secondary Events				
	Death (*n* = 18,162)	AIS (*n* = 3123)	AMI (*n* = 396)	AHS (*n* = 578)	No Event (*n* = 19,377)
**Age, years**	71.6 ± 9.7	65.6 ± 10.6	59.9 ± 11.7	65.4 ± 10.3	57.3 ± 10.5
**Male, *n* (%)**	10,528 (58.0)	2039 (65.3)	273 (68.9)	369 (63.8)	14,799 (76.4)
**Diabetes, *n* (%)**	6198 (34.1)	1172 (37.5)	135 (34.1)	194 (33.6)	5277 (27.2)
**Hypertension, *n* (%)**	16,715 (92.0)	3100 (99.3)	391 (98.7)	571 (98.8)	19,236 (99.3)
**Dyslipidemia, *n* (%)**	11,537 (63.5)	2644 (84.7)	336 (84.8)	464 (80.3)	17,662 (91.1)
**SES, *n* (%)**					
**Low**	5724 (31.5)	1009 (32.3)	119 (30.1)	166 (28.7)	4953 (25.6)
**Middle**	5357 (29.5)	1005 (32.2)	123 (31.1)	181 (31.3)	6543 (33.8)
**High**	7081 (39.0)	1109 (35.5)	154 (38.9)	231 (40.0)	7881 (40.7)

Abbreviations: AHS, acute hemorrhagic stroke; AIS, acute ischemic stroke; AMI, acute myocardial infarction; SES, socioeconomic status.

Table S4. Baseline characteristics according to the secondary events in patients with initial acute hemorrhagic stroke.

Variables	Secondary Events				
	Death (*n* = 24,990)	AIS (*n* = 3676)	AMI (*n* = 2746)	AHS (*n* = 8547)	No Event (*n* = 11,453)
**Age, years**	72.5 ± 9.5	64.4 ± 10.9	63.0 ± 11.7	64.3 ± 11.1	60.6 ± 9.4
**Male, *n* (%)**	11,782 (47.1)	1974 (53.7)	249 (60.6)	3869 (45.3)	5190 (45.3)
**Diabetes, *n* (%)**	3857 (15.4)	782 (21.3)	99 (24.1)	1243 (14.5)	1566 (13.7)
**Hypertension, *n* (%)**	19,387 (77.6)	3467 (94.3)	388 (94.4)	7493 (87.7)	10,822 (94.5)
**Dyslipidemia, *n* (%)**	4610 (18.4)	1303 (35.4)	90 (46.2)	2439 (28.5)	4635 (40.5)
**SES, *n* (%)**					
**Low**	8150 (32.6)	1184 (32.2)	126 (30.7)	2636 (30.8)	3346 (29.2)
**Middle**	7577 (30.3)	1224 (33.3)	146 (35.5)	2764 (32.3)	3954 (34.5)
**High**	9263 (37.1)	1268 (34.5)	139 (33.8)	3147 (36.8)	4153 (36.3)

Abbreviations: AHS, acute hemorrhagic stroke; AIS, acute ischemic stroke; AMI, acute myocardial infarction; SES, socioeconomic status.
